# Structure of the Brain of the Smallest Coleoptera

**DOI:** 10.1134/S1607672922040068

**Published:** 2022-08-29

**Authors:** A. A. Makarova, A. A. Polilov

**Affiliations:** Moscow State University, Moscow, Russia

**Keywords:** brain, miniaturization, Ptiliidae, *Scydosella*

## Abstract

The structure of the brain of the smallest coleopteran, *Scydosella musawasensis* Hall, 1999, is described for the first time. As in other extremely small beetles, the brain of *S. musawasensis* displays signs of miniaturization: displacement to the thorax, compactization, and a small number and size of the neurons. The body size of the studied smallest beetle is similar to that of the minute hymenopteran *Megaphragma*, which has a nearly anucleate nervous system. However, the structure of the brain of the studied smallest beetle is similar to that of large representatives of the order and is characterized by a high number of nuclei in the brain and a significant volume of the cell body rind. The neuropil of *S. musawasensis* occupies 60% of the brain volume, confirming the neuropilar constant rule.

Body size is an important characteristic that largely determines the species morphology, physiology, and biology [1]. Panarthropoda achieved the greatest success in miniaturization [2–6]. It was shown that an extreme reduction in the body size in insects has a critical effect on the anatomy, contributing to significant changes or transformation of all, or almost all, systems of organs [4]. Despite a high degree of conservatism [7], the miniaturization of the nervous system in insects is accompanied by oligomerization and concentration of ganglia, asymmetry, and a significant decrease in the number and size of neurons [4, 8]. In extreme cases of miniaturization, cellular reductions are observed [4].

Cell enucleation is a rare phenomenon in the animal kingdom. The best known anucleate cells are human erythrocytes [9]. Enucleation was also described for blood cells of mammals [10], salamanders [11], fishes [12], and insects [13]. Anucleate cells were also found in the majority of the organ systems of sexually mature dwarf male cyclophores [14]. However, the anucleate neurons were first described for three species of the genus *Megaphragma* [15, 16]. After the publication of the data on the lysis of over 97% of the nuclei of neurons at the last stages of pupal development in representatives of the genus *Megaphragma*, a question arose of whether this phenomenon is observed in other groups of the smallest insects.

Allometric analysis of the ratio of the neuropil volume to the brain volume showed that, in the majority of insects, the relative neuropil volume changes strictly isometrically [17]. Based on this analysis, the neuropilar constant rule was formulated, according to which the relative neuropil volume of the insect brain averages 60% and remains constant regardless of the body size. This rule is confirmed in the majority of insects, except for the smallest *Megaphragma*, which lacks the cell body rind in the classical sense. A question arose whether the neuropilar constant is retained in the smallest beetles?

This is the first paper to describe the brain structure of the smallest beetle *Scydosella musawasensis* Hall, 1999. The body size of these beetles is only 325 µm and today they are the smallest free-living insects in the world [18].

Adult *Scydosella musawasensis* Hall, 1999 (Ptiliidae) collected in the Chicaque National Park, Colombia in 2015 were the study object [18]. Their body length is 325–352 µm. The brain structure was studied as described earlier [16]. On the basis of a complete series of histological sections, three-dimensional brain reconstructions were made using the Bitplane Imaris and Blender software. On the basis of the three-dimensional models, the volumes of the body, brain, neuropil, and cell body rind were calculated. The linear dimensions of the cell bodies were calculated on the basis of the area of the cell body rind and the number of nuclei on ten equidistant sections evenly covering the entire brain. The number of cells was calculated on the basis of the cell body rind volume and the average size of the cell bodies.

The brain of *S. musawasensis* is elongated, in contrast to the brain of the related *Nanosella* sp., which is round and compact [8], with slightly asymmetrical posterior protocerebral lobes. The posterior protocerebral part of the brain is represented exclusively by the cell body rind and, similarly to the brain structure in other microcoleopterans, is shifted to the prothoracic region [8] (Fig. 1). The optical lobes are represented by four neuropils: lamina ganglionaris, medulla externa, lobula, and lobula plate (Fig. 2). The medulla is large, elongated in the anteroposterior plane, forms long protrusions of the anterior part of the brain (Fig. 1), and exceeds all other optic neuropils in volume. The antennal lobes are large, elongated, and have a pronounced glomerular structure. In the central complex region, fan-shaped and ellipsoidal bodies are identified. Due to the small size of the object, identification of the protocerebral bridge and mushroom bodies is hampered.

**Fig. 1.**
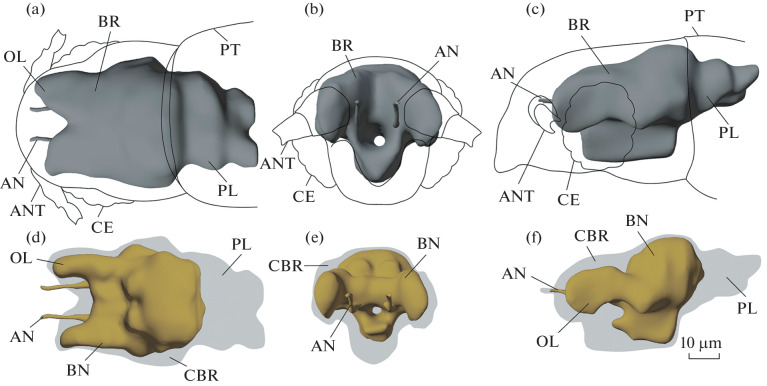
Brain structures of *Scydosella musawasensis* (Ptiliidae), 3D reconstruction: (a, d) top view; (b, e) front view; (c, f) side view. (a–c) General topography of the brain; (d–f) ratio of the cell body rind and neuropil. Designations: AN—antennal nerve, ANT—antennas, CBR—cell body rind, BR—brain; BN—brain neuropil, OL—protruding optic lobes of the brain, PT—prothorax, PL—posterior protocerebral lobes, CE—compound eyes.

**Fig. 2.**
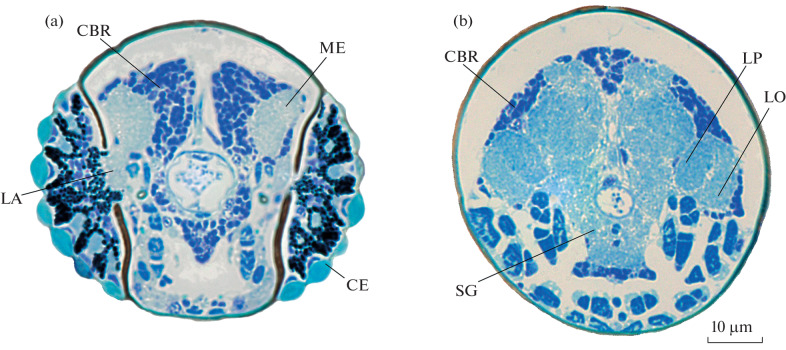
Brain structure of *Scydosella musawasensis* (Ptiliidae), histology: (a) transverse section through compound eyes; (b) cross section of the posterior part of the head capsule. Designations: CBR—cell body rind, LA—lamina, ME—medulla, LO—lobula, LP—lobular plate, SG—suboesophageal ganglion, CE—compound eyes.

The absolute brain volume is 0.045 nL, which is 2–3 times less than in the smallest of the previously studied beetles [17] and is similar to the brain volume of one of the smallest insects, *Megaphragma amalphitanum* (0.041 nL). Although the body size and absolute brain volume of *S. musawasensis* are similar to those of *M. amalphitanum*, *S. musawasensis* has a significant cell body rind volume (Fig. 2), which accounts for approximately 42% of the total brain volume. Thus, the neuropilar constant rule, according to which insects have a constant ratio of volumes of the neuropil (60% of the brain volume) and cell body rind (40% of the brain volume), is confirmed [17]. At comparable body sizes, *S. musawasensis* has a significant amount of the cell body rind, whereas adult *M. amalphitanum* are characterized by an almost complete loss of up to 97% of neuronal nuclei at the late stages of pupal development. It is likely that the phenomenon of lysis of neuronal nuclei, which explains the violation of the neuropilar constant in the case of *Megaphragma*, is absent in miniature beetles.

The brain of *S. musawasensis* contains approximately 9500 cells with an average diameter of approximately 1.25 µm. A similar number of neurons in the brain was found in the smallest of the previously studied beetles, *Nanosella* sp. (body length approximately 400 μm, about 8000 cells in the brain) [4].

Despite the small number and size of neurons, *S. musawasensis* has a large relative brain volume, similarly to the majority of other microinsects, except for some lines of *Trichogramma* [19] and *Nasonia* [20]. The relative brain volume of *S. musawasensis* is significantly larger than that of large beetles [4] and is approximately 4%.

Thus, miniaturization in different groups of insects has different effects on the brain structure. Coleoptera and thrips have a wide neck, which makes the head less mobile, and also allows the brain not to be limited to the head capsule and partially shift to the thoracic region [4]. In Hymenoptera, on the contrary, due to the thin and narrow neck, the head is more mobile, and the brain is strictly limited to the head capsule and extremely compact [4]. Extreme miniaturization in *Megaphragma* led to extreme adaptations (reductions of the nuclei and bodies of neurons), but did not affect the cellular structure of the brain in the smallest *Scydosella*.

Despite the extreme conservatism of the brain structure, morphological adaptations to miniaturization may significantly differ in insects with an extremely small body size.
