# Erratum to: **Structure of the Brain of the Smallest Coleoptera**

**DOI:** 10.1134/S1607672955340036

**Published:** 2023-02-14

**Authors:** A. A. Makarova, A. A. Polilov

**Affiliations:** Moscow State University, 119991 Moscow, Russia

The article “Structure of the Brain of the Smallest Coleoptera,” written by A. A. Makarova and A. A. Polilov and presented by Academician V. V. Rozhnov, was originally published electronically in Springer-Link on August 29, 2022 without Open Access. After publication in volume 505, issue 1, pages 166–169 the authors decided to make the article an Open Access publication. Therefore, the copyright of the article has been changed to © The Author(s) 2022 and the article is forthwith distributed under the terms of a Creative Commons Attribution 4.0 International License (http://creativecommons.org/licenses/by/4.0/, CC BY), which permits use, duplication, adaptation, distribution and reproduction of a work in any medium or format, as long as you cite the original author(s) and publication source, provide a link to the Creative Commons license, and indicate if changes were made.

